# A disparate role of RP11-424C20.2/UHRF1 axis through control of tumor immune escape in liver hepatocellular carcinoma and thymoma

**DOI:** 10.18632/aging.102197

**Published:** 2019-08-23

**Authors:** Jue Yang, Yongqiang Zhang, Hui Song

**Affiliations:** 1 The Key Laboratory of Endemic and Ethnic Diseases, Guizhou Medical University, Ministry of Education, Guiyang 550004, China; 2 The Key Laboratory of Medical Molecular Biology, Guizhou Medical University, Guiyang 550004, China; 3 The State Key Laboratory of Functions and Applications of Medicinal Plants, Guizhou Medical University, Guiyang 550014, China; 4 Department of Urology, Guizhou Province People’s Hospital, Guiyang 550002, China

**Keywords:** pseudogene, UHRF1, immune escape, PD-L1, CLTA-4

## Abstract

The immune system is critical in modulating cancer progression. Pseudogenes are a special type of long non-coding RNAs that regulate different tumorigenic processes. However, the potential roles of pseudogenes in tumor-immune interaction remain largely unclear. Here, we reported that pseudogene RP11-424C20.2 and its parental gene UHRF1 were frequently up-regulated and positively correlated in liver hepatocellular carcinoma (LIHC) and thymoma (THYM), but associated with distinct clinical outcomes. We further found that RP11-424C20.2 may act as a competing endogenous RNA (ceRNA) to increase UHRF1 expression through sponging miR-378a-3p. Functional enrichment analysis showed a strong association of UHRF1 with immune-related biological processes. We also observed that UHRF1 expression significantly correlated with immune infiltration, and different types of tumor-infiltrating immune cells displayed different impacts on clinical outcomes. Furthermore, UHRF1 expression in LIHC and THYM showed an opposite correlation with biomarkers from monocyte, dendritic cell, Th1 and T cell exhaustion. Mechanism investigations revealed that RP11-424C20.2/UHRF1 axis regulated immune escape of LIHC and THYM at least partly through IFN-γ-mediated CLTA-4 and PD-L1 pathway. These findings demonstrate a disparate role of RP11-424C20.2/UHRF1 axis in LIHC and THYM via regulating immune infiltrates, and also indicate a therapeutic value for UHRF1 inhibitors in combination with anti-PD-L1/CLTA-4 blockade.

## INTRODUCTION

Tumor initiation is a complex process involved in intracellular gene mutations and intercellular interaction with tumor microenvironment. Multiple molecules and pathways participate in tumor development and progression. A certain cancer may be initiated with different gene alterations but one gene dysregulation can bring distinct clinical outcomes. For instance, miR-374a has been reported to function as an oncogene during tumor pathogenesis in breast cancer [1]. Meanwhile, several studies also suggest a role of suppressor gene in nasopharyngeal carcinoma and lung adenocarcinoma [2, 3]. Even in the same tumor, miR-374a can play a dual role to regulate tumorigenesis of non-small-cell lung cancer via interacting with different target genes [4]. These molecular polymorphisms and alterations require precision oncology based on individual difference.

Recent years, great advancement has been achieved in immunotherapy especially with the introduction of checkpoint blockers into cancer treatment, such as antibodies blocking PD-1/PD-L1 and CTLA-4 [5]. However, the achievements of cancer immunotherapy are eclipsed by low response rates to metastatic patients and more importantly, often follow with adverse effects [6]. To improve the effectiveness, it is reasonable to develop a highly “personalized” immunotherapy for common cancers for each patient. Public large-scale cancer omics data, such as The Cancer Genome Atlas (TCGA), provide us diverse clinical feathers and molecular data of cancer patients, which will largely broaden our horizon about the underlying mechanism of tumorigenesis.

Pseudogenes are a special type of long non-coding RNAs that regulate their parental genes or unrelated genes expression through interacting with diverse DNAs, RNAs or proteins [7]. Recent advances have established that pseudogenes play important roles in several biological processes relevant to the development of cancer [8]. High level of PDIA3P1 was associated with poor prognosis of liver hepatocellular carcinoma (LIHC) and promoted cell proliferation and metastasis through inhibiting the p53 pathway [9]. In addition, pseudogene-encoded proteins presented on surface of malignant cells can provide new antigens which are crucial for immune system recognition against human cancer [10, 11]. To data, the role of pseudogenes in tumor-immune interaction is still limited.

Previously, we reported a pseudogene RP11-424C20.2 was frequently up-regulated in human cancers and high levels of RP11-424C20.2 always predicted a worse outcome [12]. Its parental gene UHRF1 is a key epigenetic regulator through coordinating DNA methylation and histone modifications [13–15]. UHRF1 is up-regulated in various human cancers and predicts poor prognosis [16]. Recent investigations revealed that highly expressed UHRF1 promoted apoptotic escape via silencing tumor suppressor genes [17, 18]. Up-regulated UHRF1 was broadly implicated in tumor progression, including cell proliferation, metastasis and chemoresistance [19–21]. However, the regulatory relationship between RP11-424C20.2 and UHRF1 in tumor progression has not been elucidated. In this study, we found that RP11-424C20.2 expression was strongly correlated with UHRF1 and RP11-424C20.2/UHRF1 axis functioned as a disparate role in LIHC and thymoma (THYM) through regulating immune infiltration.

## RESULTS

### Up-regulated RP11-424C20.2 and UHRF1 are significantly associated with prognosis of cancer patients

**Figure 1:**
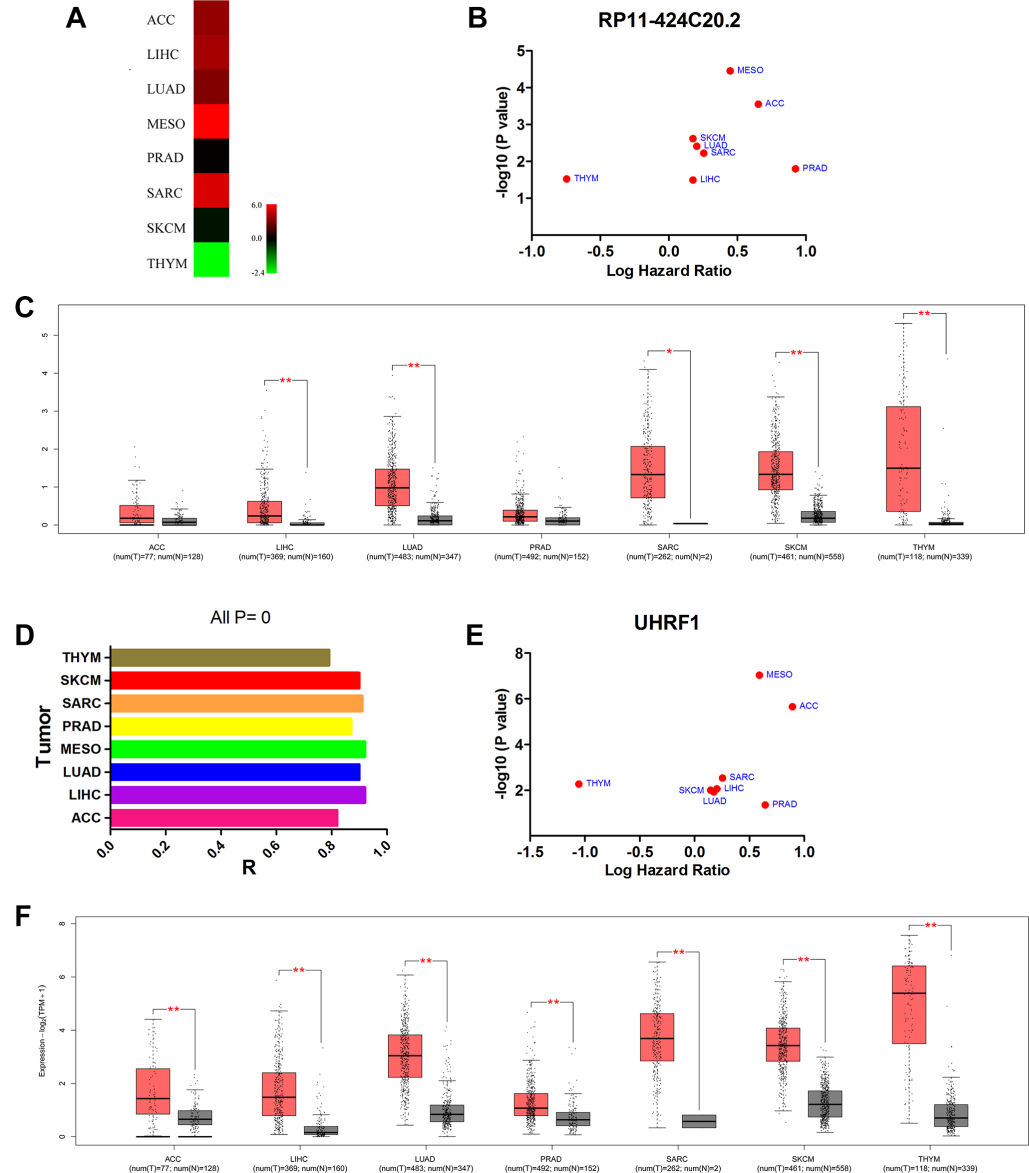
**Up-regulated RP11-424C20.2 and UHRF1 are significantly associated with prognosis of cancer patients.** (**A**) RP11-424C20.2 was dysregulated in human cancer identified using dreamBase. (**B**) Prognostic values of RP11-424C20.2 analyzed with GEPIA. (**C**) RP11-424C20.2 expression was validated using GEPIA. (**D**) Correlation analysis between RP11-424C20.2 and UHRF1 using GEPIA. (**E**) Prognostic values of UHRF1 analyzed with GEPIA. (**F**) UHRF1 expression was evaluated by GEPIA.

In the previous study, we found that RP11-424C20.2 was dysregulated in 32 types of human cancer and high expression levels of RP11-424C20.2 predicted worse overall survival of patients with adrenocortical carcinoma (ACC), LIHC, lung adenocarcinoma (LUAD), mesothelioma (MESO), prostate adenocarcinoma (PRAD), sarcoma (SARC) or skin cutaneous melanoma (SKCM) but better outcome of patients with THYM (Figure 1A and 1B). RP11-424C20.2 expression levels in 7 types of human cancer from TCGA were validated using GEPIA and found RP11-424C20.2 was significantly up-regulated in LIHC, LUAD, SARC, SKCM and THYM (Figure 1C). To further investigate the potential roles of RP11-424C20.2 in the progression of human cancer, we first blasted its sequence in the human genome and found that there was a 99% similarity between RP11-424C20.2 and its parental gene UHRF1 (NM_001048201.2) (Supplementary Figure 1). Pearson correlation analysis revealed a strong positive relationship between RP11-424C20.2 and UHRF1 in all 8 types of cancer (Figure 1D). We observed an increased mRNA expression of UHRF1 in ACC, LIHC, LUAD, MESO, PRAD, SARC, SKCM and THYM compared with normal control (Figure 1F). Consistent with RP11-424C20.2, UHRF1 was also significantly associated with prognosis in the 8 types of cancer (Figure 1E). These results suggest that RP11-424C20.2 in THYM and other types of cancer may play a disparate role through regulating its parental gene UHRF1 expression.

### RP11-424C20.2 functions as a sponge of miR-378a-3p to regulate UHRF1 expression

**Figure 2:**
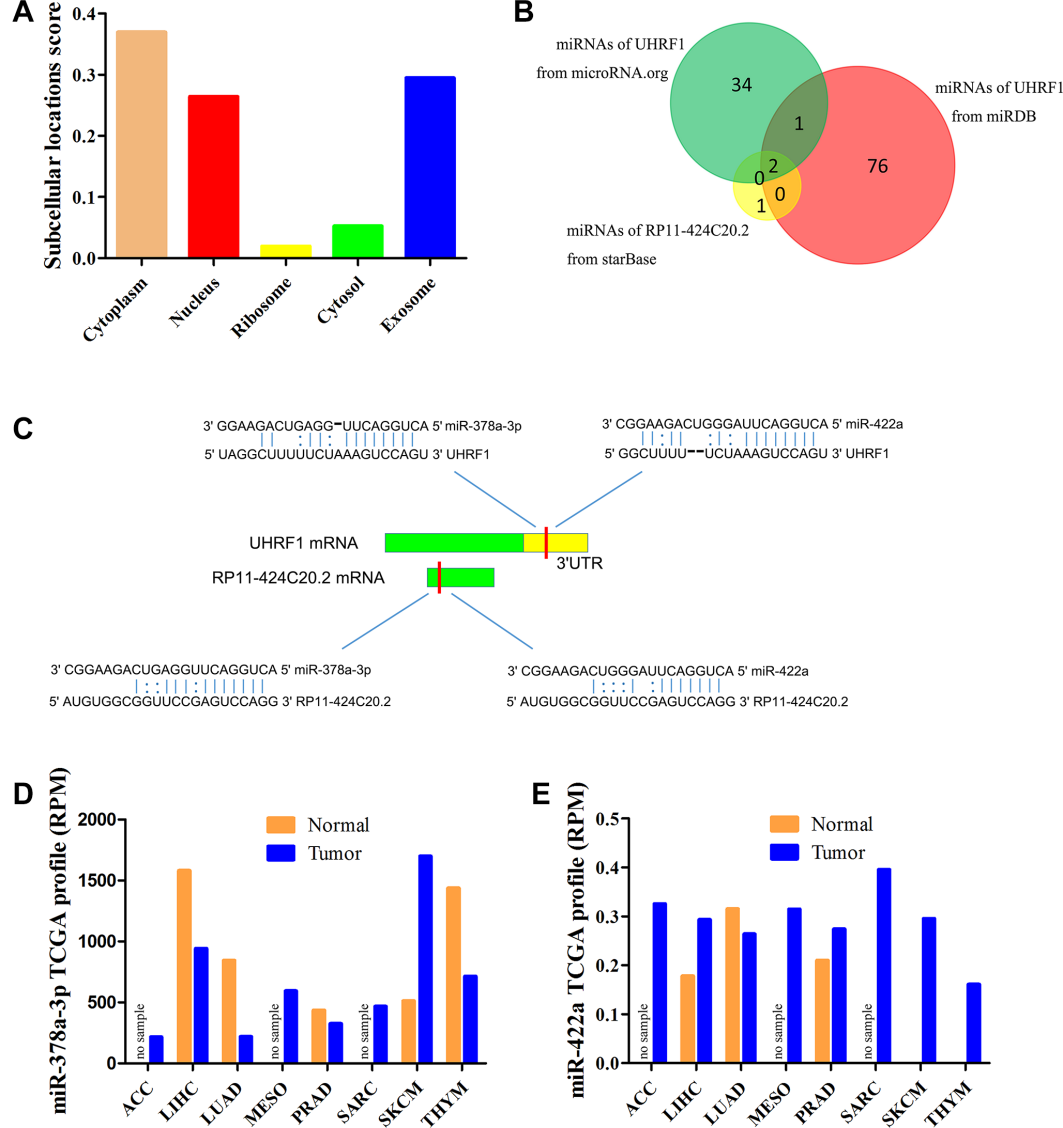
**miR-378a-3p is identified as candidate miRNA.** (**A**) Prediction of cellular localization for RP11-424C20.2 using lncLocator. (**B**) Bioinformatics analysis of candidate miRNAs for RP11-424C20.2 and UHRF1. (**C**) Base pairing between miR-378a-3p and miR-422a and the putative target site in the RP11-424C20.2 and UHRF1 3’UTR predicted by starBase v2.0 and microRNA.org, respectively. (**D**) miR-378a-3p expression in TCGA samples. (**E**) miR-422a expression in TCGA samples.

As a special type of long non-coding RNAs, cellular localization of pseudogenes determined the underlying mechanisms. lncLocator predicted that RP11-424C20.2 mainly located in the cytoplasm but also distributed in the exosome and nucleus (Figure 2A). The result indicates that RP11-424C20.2 regulates UHRF1 expression more likely via competing endogenous RNA (ceRNA) mechanism. Bioinformatics tools prediction identified miR-378 and miR-422a as candidate miRNAs (Figure 2B). We further analyzed their expression levels using miR_Path from TCGA samples. Our results showed that miR-378a-3p was down-regulated in LIHC, LUAD, PRAD and THYM but up-regulated in SKCM (Figure 2C). miR-422a was up-regulated in LIHC, PRAD, SKCM and THYM (Figure 2D). These data indicate that RP11-424C20.2 may act as ceRNA to promote UHRF1 expression through sponging miR-378a-3p.

### UHRF1 expression is correlated with immune infiltration in LIHC and THYM

**Figure 3:**
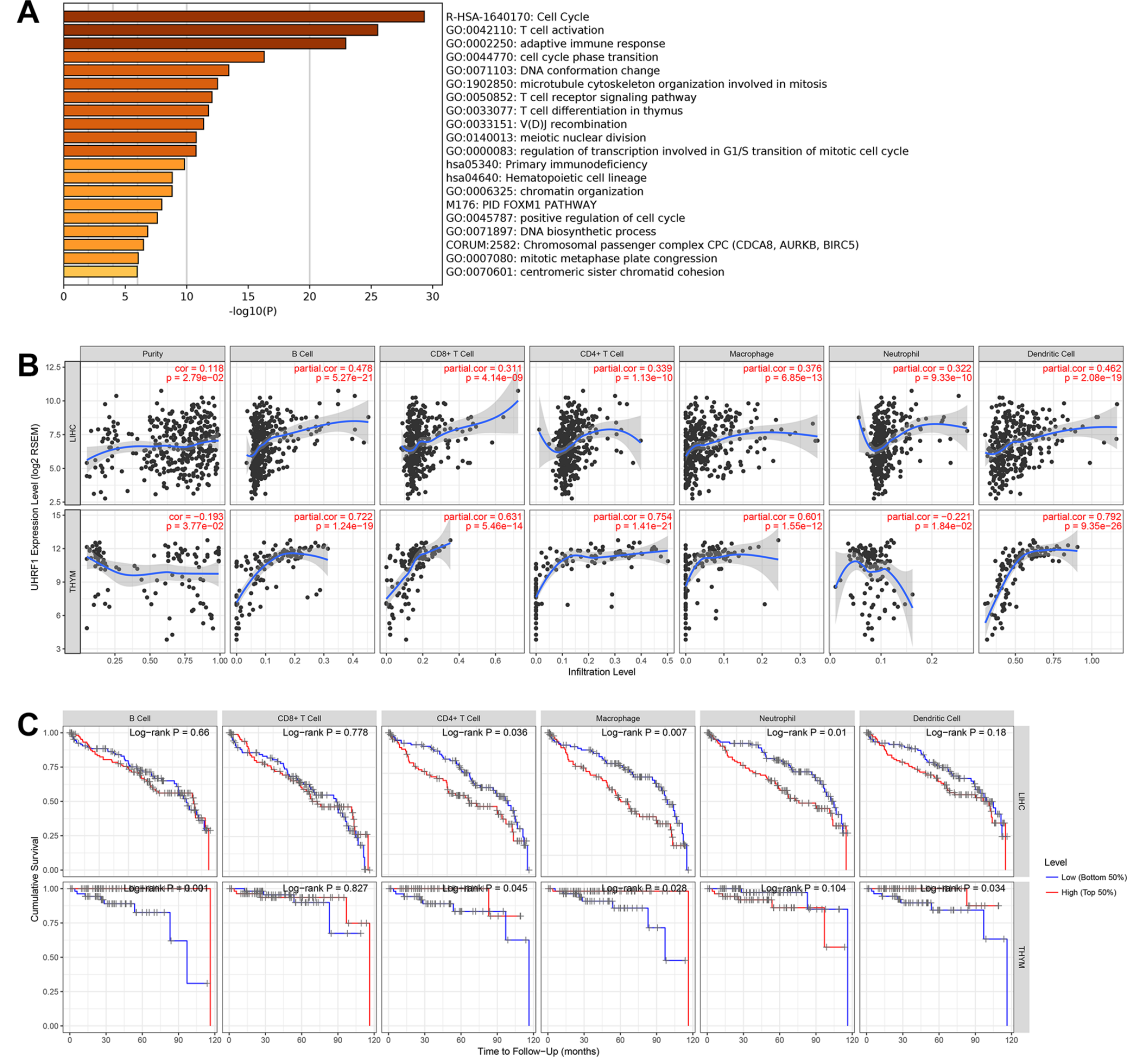
**UHRF1 expression is correlated with immune infiltration in LIHC and THYM.** (**A**) GO and KEGG enrichment analysis of UHRF1-related genes. (**B**) Correlation of UHRF1 expression with immune infiltration analyzed using the “Gene” module in TIMER. (**C**) Kaplan–Meier plots for immune infiltrates and overall survival of LIHC and THYM were visualized using the “Survival” module in TIMER.

To further explore potential function of RP11-424C20.2 in the cancer development, we performed gene ontology (GO) and Kyoto Encyclopedia of Genes and Genomes (KEGG) pathway enrichment analysis of the top 200 correlated genes of UHRF1 in the 8 types of cancer. The most significant enriched term was cell cycle (R-HSA-1640170). Interestingly, we also observed that UHRF1 was closely related to immune-associated biological processes, such as T cell activation (GO:0042110), adaptive immune response (GO:0002250), T cell receptor signaling pathway (GO:0050852) and T cell differentiation in thymus (GO:0033077) (Figure 3A). Thus, RP11-424C20.2-UHRF1 axis may be involved in the interaction between tumor and immune response.

To test this hypothesis, we analyzed the relationship between UHRF1 expression and immune infiltration levels in 8 types of cancer using TIMER. In LIHC and THYM, our results showed a strongest correlation of UHRF1 expression and immune infiltration level, including tumor purity, B cell, CD8+ cell, CD4+ cell, macrophage, neutrophil and dendritic cell (Figure 3B and Supplementary Figure 2). We further evaluated the impact of tumor-infiltrating immune cells on clinical outcomes of patients with LIHC or THYM using the “Survival” module in TIMER. As shown in Figure 3C, high levels of CD4+ cell, macrophage and neutrophil predicted better outcome for LIHC patients with survival time within 24 months (P=0.036, P=0.007 and P=0.01, respectively). However, high levels of B cell, CD4+ cell, macrophage and dendritic cell predicted worse outcome for THYM patients with survival time within 120 months (P=0.001, P=0.045, P=0.028 and P=0.034, respectively). These findings demonstrate that RP11-424C20.2/UHRF1 axis affects clinical outcomes of patients with LIHC and THYM through regulating tumor-infiltrating immune cell level.

### Correlation analysis between UHRF1 expression and immune marker sets

To validate the association of UHRF1 with immune infiltration, we further evaluated the correlation between UHRF1 expression and 57 biomarkers from 16 subtypes of tumor-infiltrating immune cells in LIHC and THYM (Table 1). Results showed a significant correlation in both LIHC and THYM, occupied 35/57 and 38/57 respectively. For biomarkers of CD8+ T cell and T cell (general), we observed a strongly positive correlation with UHRF1 expression in both LIHC and THYM. Surprisingly, results revealed an obvious opposite tendency of biomarkers from monocyte, dendritic cell, Th1 and T cell exhaustion between LIHC and THYM. Distinguishingly, UHRF1 expression significantly correlated with biomarkers of Tfh (T follicular helper) cells in LIHC and biomarkers of M1 and M2 macrophage in THYM. We further validated the difference of biomarkers from monocyte, dendritic cell, Th1 and T cell exhaustion between LIHC and THYM using GEPIA. Similarity to TIMER, GEPIA analysis also showed an opposite tendency for these biomarkers in LIHC and THYM (Supplementary Table 1). These results indicate that different antigen presentations of tumor-infiltrating immune cells may also contribute to the distinct clinical outcomes for RP11-424C20.2/UHRF1 axis in LIHC and THYM.

**Table 1:** Correlation analysis between UHRF1 and biomarkers of immune cells using TIMER.

**Description**	**Gene markers**	**LIHC**		**THYM**	
		**Cor**	**P**	**Cor**	**P**
CD8+ T cell	CD8A	0.237	***	0.823	***
	CD8B	0.231	***	0.721	***
T cell (general)	CD3D	0.31	***	0.713	***
	CD3E	0.26	***	0.799	***
	CD2	0.282	***	0.736	***
B cell	CD19	0.281	***	-0.17	0.069
	CD79A	0.232	***	0.532	***
Monocyte	CD86	0.318	***	-0.564	***
	CD115 (CSF1R)	0.153	*	-0.61	***
TAM	CCL2	0.075	0.165	-0.238	0.010
	CD68	0.093	0.084	-0.279	*
	IL10	0.215	***	-0.072	0.447
M1 Macrophage	INOS (NOS2)	-0.107	0.047	-0.339	**
	IRF5	0.169	*	-0.478	***
	COX2 (PTGS2)	0.112	0.038	-0.521	***
M2 Macrophage	CD163	0.032	0.552	-0.353	**
	VSIG4	0.09	0.095	-0.454	***
	MS4A4A	0.061	0.261	-0.09	0.341
Neutrophils	CD66b (CEACAM8)	0.054	0.315	0.336	**
	CD11b (ITGAM)	0.335	***	-0.216	0.020
	CCR7	0.164	*	0.216	0.020
Natural killer cell	KIR2DL1	-0.113	0.036	-0.197	0.035
	KIR2DL3	0.127	0.018	-0.23	0.014
	KIR2DL4	0.162	*	-0.434	***
	KIR3DL1	-0.022	0.678	-0.294	*
	KIR3DL2	0.072	0.185	0.03	0.753
	KIR3DL3	0.061	0.257	-0.007	0.945
	KIR2DS4	0.004	0.948	0	0.998
Dendritic cell	HLA-DPB1	0.178	**	-0.337	**
	HLA-DQB1	0.185	**	-0.149	0.112
	HLA-DRA	0.193	**	-0.413	***
	HLA-DPA1	0.178	**	-0.398	***
	BDCA-1 (CD1C)	0.103	0.057	0.803	***
	BDCA-4 (NRP1)	0.016	0.768	-0.417	***
	CD11c (ITGAX)	0.32	***	-0.477	***
Th1	T-bet (TBX21)	0.061	0.258	-0.274	*
	STAT4	0.276	***	-0.16	0.088
	STAT1	0.286	***	-0.48	***
	IFN-γ (IFNG)	0.287	***	-0.509	***
	TNF-α (TNF)	0.295	***	-0.489	***
Th2	GATA3	0.246	***	0.777	***
	STAT6	-0.267	***	-0.353	**
	STAT5A	0.118	0.028	-0.294	*
	IL13	0.119	0.027	-0.087	0.354
Tfh	BCL6	-0.142	*	0.092	0.329
	IL21	0.191	**	-0.163	0.082
Th17	STAT3	-0.123	0.022	-0.582	***
	IL17A	0.044	0.419	-0.204	0.029
	FOXP3	0.164	*	-0.444	***
	CCR8	0.433	***	0.433	***
	STAT5B	-0.191	**	0.258	*
	TGFβ (TGFB1)	0.234	***	-0.123	0.190
T cell exhaustion	PD-1 (PDCD1)	0.374	***	0.59	***
	CTLA4	0.38	***	-0.407	***
	LAG3	0.259	***	-0.535	***
	TIM-3 (HAVCR2)	0.354	***	-0.403	***
	GZMB	0.051	0.342	-0.065	0.493

*P<0.01, **P<0.001, ***P<0.0001

### PD-L1 and CTLA-4 are potential downstreams of RP11-424C20.2/UHRF1 axis

PD-1/PD-L1 and CTLA-4 are key immune checkpoint molecules that elicit an immune response against tumor. Next, we explored their relationship with UHRF1 expression in LIHC and THYM. Results showed that UHRF1 expression levels were positively correlated with PD-1 expression in both LIHC and THYM (Supplementary Figure 3). However, there was a highly reverse association between UHRF1 and PD-L1 or CTLA-4 in LIHC and THYM. We supposed that the different roles of RP11-424C20.2/UHRF1 axis in LIHC and THYM may result from control of PD-L1/CTLA-4 expression.

**Figure 4:**
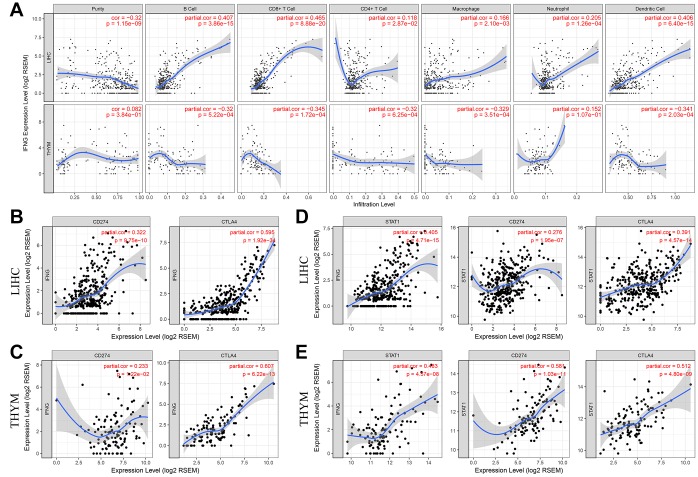
**PD-L1 and CTLA-4 are potential downstreams of RP11-424C20.2/UHRF1 axis.** (**A**) Correlation of IFN-γ expression with immune infiltration in LIHC and THYM. (**B**) and (**C**) Correlation analysis between IFN-γ expression and PD-L1 or CTLA-4 in LIHC and THYM. (**D**) and (**E**) Correlation analysis between STAT1 and IFN-γ, PD-L1 or CTLA-4 in LIHC and THYM.

IFN-γ is a cytokine that plays pivotal roles in immune response and tumor immunosurveillance [22]. As shown in Table 1, UHRF1 expression levels in LIHC and THYM was reversely correlated with IFN-γ (R=0.287, P=5.80e-08; R=-0.509, P=6.33e-09, respectively). In addition, there was an opposite tendency between IFN-γ expression and immune infiltration of B cell, CD8+ T cell, macrophage and dendritic cell in LIHC and THYM (Figure 4A). We also found that IFN-γ expression levels in both LIHC and THYM was strongly associated with CTAL-4 and PD-L1 expression (Figure 4B and 4C). These results indicate that RP11-424C20.2/UHRF1 axis may regulate CTLA-4 and PD-L1 expression through IFN-γ signaling. STAT1 is an important transitional factor, which can be activated IFN-γ [23]. To investigate the potential role of STAT1, we analyzed the relationship between STAT1 and IFN-γ, PD-L1 or CTLA-4 in LIHC and THYM. Correlation analysis of TIMER showed STAT1 was significantly associated with IFN-γ, CTLA-4 and PD-L1 in both LIHC and THYM (Figure 4D and 4E). GEPIA analysis further confirmed this association (Supplementary Table 2). These findings suggest RP11-424C20.2/UHRF1 axis regulates immune infiltration of LIHC and THYM at least partly through IFN-γ-mediated CLTA-4 and PD-L1 pathway.

## DISCUSSION

RP11-424C20.2 also known as AC112777.1, is a processed pseudogene with a length of 1423 bp and located in chromosome 12p12.2. Previous studies have established that pseudogenes can function as antisense RNAs, interference RNAs or gene competitors to affect their parental genes or unrelated genes expression [24]. Pseudogenes most likely impact their parental gene expression via ceRNA network in which pseudogene RNAs interact with their counterparts through competitively binding to common miRNAs and attenuate repression on the parental genes [25]. In this context, pseudogene PTENP1 up-regulated its parental gene PTEN expression by sponging miR-19b, miR-21, miR-193-3p and miR-200c [26–29]. In this study, we observed that RP11-424C20.2 expression was strongly correlated with its parental gene UHRF1 expression in 8 types of human cancer. In addition, our data suggest RP11-424C20.2 may function as ceRNA to up-regulate UHRF1 expression through sponging miR-378a-3p. A similar regulatory effect was recently observed in which UHRF1 was reported to be a direct target gene of miR-378 in medulloblastoma [30].

Our data also showed that up-regulated RP11-424C20.2/UHRF1 predicted poor prognosis of LIHC patients but favorable outcome of THYM patients. To investigate the reason for this distinct clinical outcomes, we analyzed the function of UHRF1-related genes. Interestingly, we found that UHRF1 was strongly correlated with immune function, especially T cells-related biological processes, indicating that RP11-424C20.2/UHRF1 axis may control the cancer progression through affecting the interactions between immune and malignant cells.

Accumulated studies have demonstrated that immune infiltrates affected the prognosis and efficacy of chemoradiotherapy and immunotherapy [31–33]. Using TIMER, we observed that UHRF1 expression was closely related to immune infiltration of LIHC and THYM. Our results also showed several types of tumor-infiltrating immune cells were significantly associated with outcomes of patients with LIHC and THYM. Previous studies reported that immune infiltration varied between and within tumors and influenced immune escape and clinical outcomes through different neoantigen presentation dysfunction affected by distinct immune microenvironments [34]. Further analysis revealed an obvious opposite tendency of neoantigens from monocyte, dendritic cell, Th1 and T cell exhaustion with UHRF1 expression between LIHC and THYM. These difference induced by RP11-424C20.2/UHRF1 axis may contribute to the change of tumor-immune microenvironment and development of LIHC and THYM.

Among different neoantigens presented on the LIHC and THYM cells, we identified PD-L1 and CTLA-4 as potential targets of RP11-424C20.2/UHRF1 axis. Through the further correlation analysis between UHRF1 and different neoantigens, we provided evidence that RP11-424C20.2/UHRF1 axis might regulate immune infiltration of LIHC and THYM at least partly through IFN-γ-mediated CLTA-4 and PD-L1 pathway.

PD-L1 and CTLA-4 are two important co-inhibitory receptors expressed on immune cells that could trigger T cell dysfunction and immune escape [35, 36]. Increasing evidence revealed that CLTA-4 and PD-L1 were regulated in several different ways, from genetic alterations and epigenetic modification to transcriptional regulation [37, 38]. Consistent with our results, IFN-γ was reported to regulate CLTA-4 and PD-L1 expression through activation of STAT1 signaling [39, 40]. In fact, IFN-γ signaling-mediated interaction of tumor-infiltrating immune cells and malignant cells is complex. IFN-γ can inhibit tumor cell proliferation and metastasis through increasing antigen presentations [41, 42]. On the other hand, IFN-γ can also suppress host immune defense via inducing expression of PD-L1 and SOCS2 [43–45]. Several studies also suggest epigenetic modifications which are probably mediated by UHRF1 activated interferon signaling through increasing endogenous retroviral elements [46–48]. In this respect, drugs such as UHRF1 inhibitors in combination with anti-PD-L1/CLTA-4 blockade will be of great therapeutic interest.

**Figure 5:**
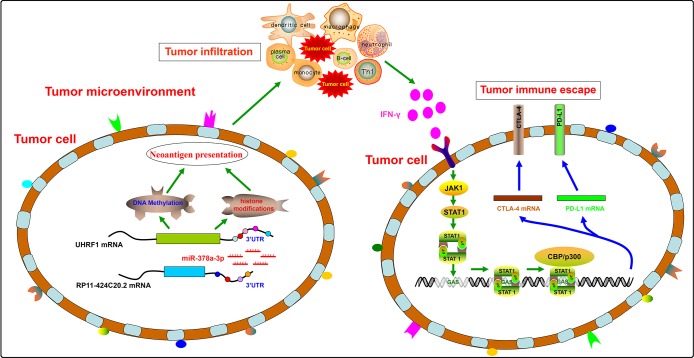
**A disparate role of RP11-424C20.2/UHRF1 axis in the progression of LIHC and THYM by regulating tumor immune escape.**

In conclusion, our results suggest a disparate role of RP11-424C20.2/UHRF1 axis in the progression of LIHC and THYM by regulating tumor immune escape which is mediated at least partly through IFN-γ-mediated CLTA-4 and PD-L1 pathway (Figure 5). Furthermore, this study demonstrates an integrated and liable method to predict the potential role of non-coding RNAs in tumor immune based on public omics data.

More importantly, we here may also provide novel therapeutic targets for cancer treatment to optimize current immunotherapy.

## MATERIALS AND METHODS

### Gene expression analysis

RP11-424C20.2 expression levels in human cancer were obtained from dreamBase, in which an integrated analysis of pseudogenes was performed for the transcriptional regulation, expression profiles and functional mechanisms [49]. Then, RP11-424C20.2 expression levels were validated using GEPIA from TCGA samples [50]. mRNA expression level of UHRF1 was analyzed by GEPIA.

### Prognostic value analysis

The relationship between gene expression level of RP11-424C20.2 or UHRF1 and overall survival of patients were analyzed using GEPIA. Kaplan-Meier plots for immune infiltrates (B Cell, CD8+ T Cell, CD4+ T Cell, Macrophage, Neutrophil and Dendritic Cell) and overall survival of patients with LIHC or THYM were visualized using the “Survival” module in TIMER and corrected for tumor purity [51]. For both GEPIA and TIMER, 50% group cutoff value, log-rank test, the Cox proportional hazard ratio and the 95% confidence interval were used for analysis. A p-value less than 0.05 was considered statistically significant.

### RP11-424C20.2 cellular localization prediction

RP11-424C20.2 sequence was obtained from UCSC [52] and its cellular localization was analyzed by its sequence using lncLocator based on a stacked ensemble classifier [53]. To data, five subcellular localizations of long non-coding RNAs, including cytoplasm, nucleus, cytosol, ribosome and exosome, can be predicated using lncLocator.

### Candidate miRNAs analysis

Potential binding miRNAs of RP11-424C20.2 and UHRF1 3’UTR were predicted using starBase v2.0 [54], microRNA.org [55] and miRDB [56] and then analyzed using Venn diagram. Expression levels of candidate miRNAs were assessed using miR_path from TCGA samples [57].

### Correlation analysis of gene expression

Correlation between RP11-424C20.2 and UHRF1 in 8 types of human cancer (ACC, LIHC, LUAD, MESO, PRAD, SARC, SKCM and THYM) or UHRF1 and 57 biomarkers from 16 tumor-infiltrating immune cells in LIHC and THYM was analyzed using GEPIA and “Correlation” module in TIMER, respectively. For GEPIA Pearson’s correlation analysis, the non-log scale for calculation and the log-scale axis for visualization were used. For TIMER Spearman’s correlation analysis, correlation was adjusted by tumor purity.

### GO and KEGG enrichment analysis

Top 200 UHRF1-associated genes in 8 types of human cancer (ACC, LIHC, LUAD, MESO, PRAD, SARC, SKCM and THYM) were obtained from GEPIA. Functional enrichment analysis of these genes was performed using Metascape [58]. Heat map of top 20 enriched terms was colored by P-values.

### Correlation of UHRF1 expression with immune infiltration analysis

Spearman’s correlation between UHRF1 expression and immune infiltration level in 8 types of human cancer (ACC, LIHC, LUAD, MESO, PRAD, SARC, SKCM and THYM) was visualized using “Gene” module in TIMER. The correlation was adjusted by tumor purity.

## Supplementary Material

Supplementary Figures

Supplementary Tables
