# Supplementary material for: A disparate role of RP11-424C20.2/UHRF1 axis through control of tumor immune escape in liver hepatocellular carcinoma and thymoma
Source: Aging (Albany NY). 2019 Aug 23;11(16):6422–39. doi: 10.18632/aging.102197 (PMC6738438; doi:10.18632/aging.102197)
Supplement: Supplementary Figures [file aging-11-102197-s002.pdf]

SUPPLEMENTARY FIGURES

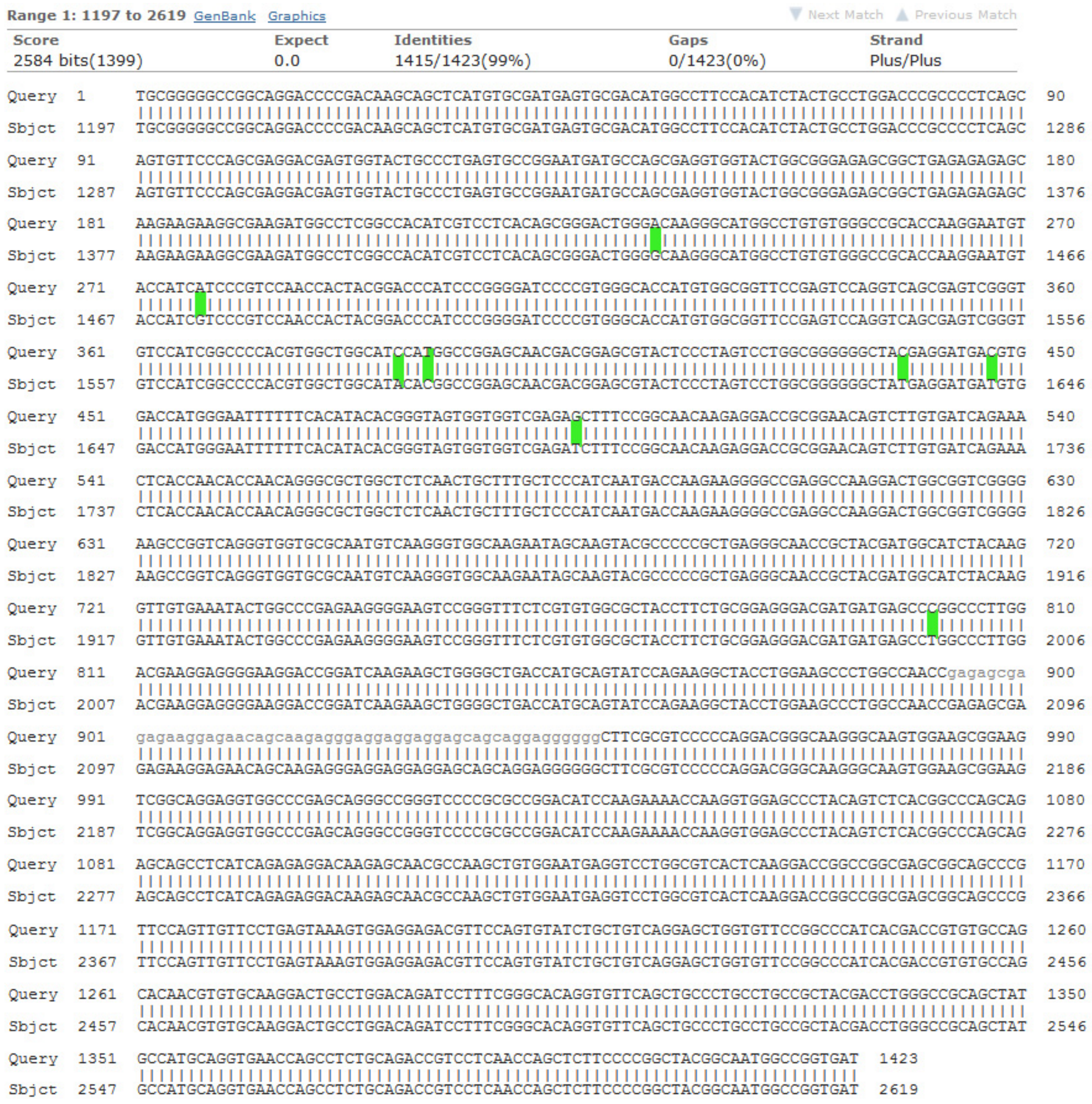

Supplementary Figure 1. Sequence similarity between RP11-424C20.2 and its parental gene UHRF1 (NM_001048201.2).

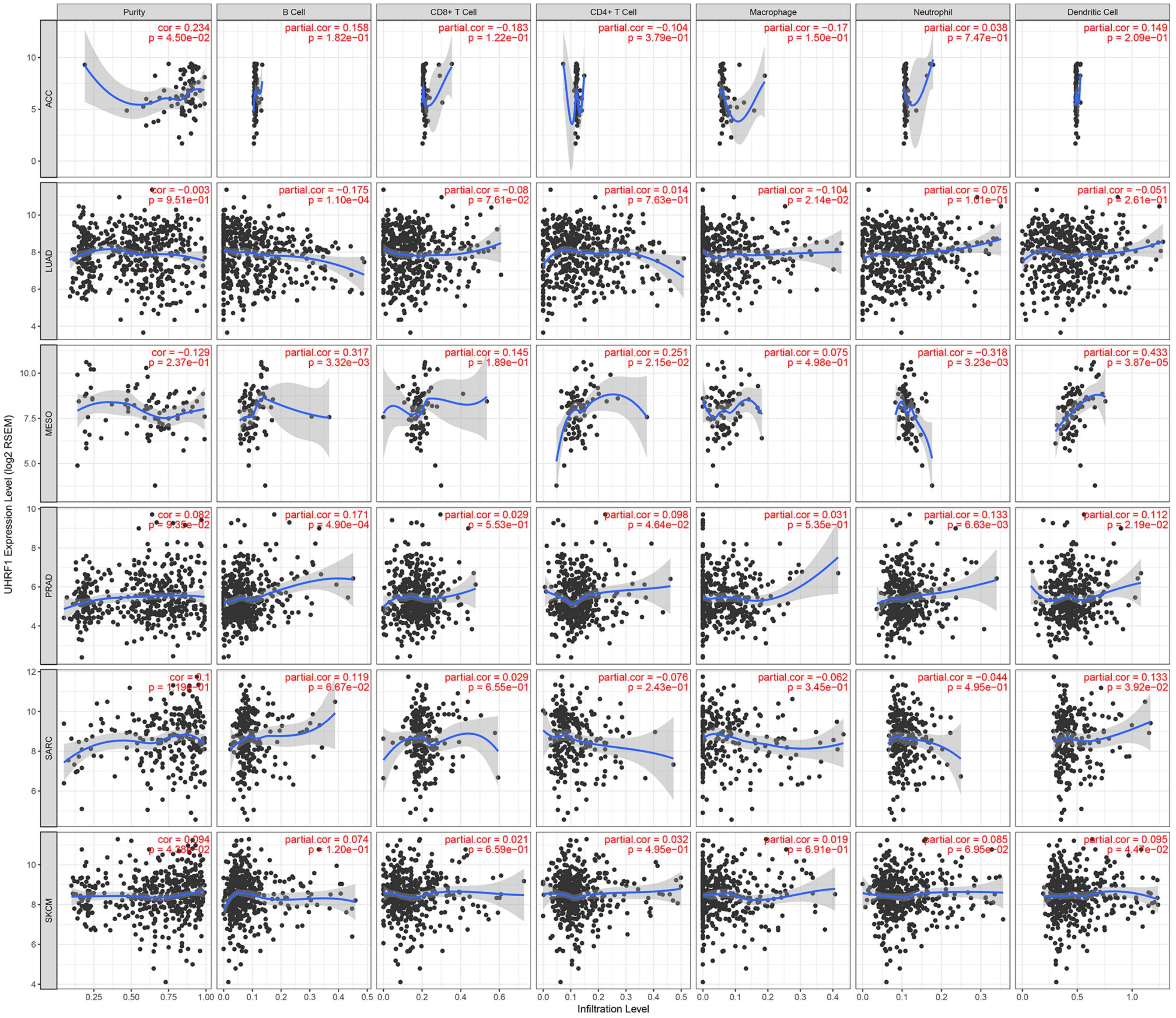

Supplementary Figure 2. Correlation of UHRF1 expression with immune infiltration in ACC, LUAD, MESO, PRAD, SARC and SKCM analyzed using the “Gene” module in TIMER.

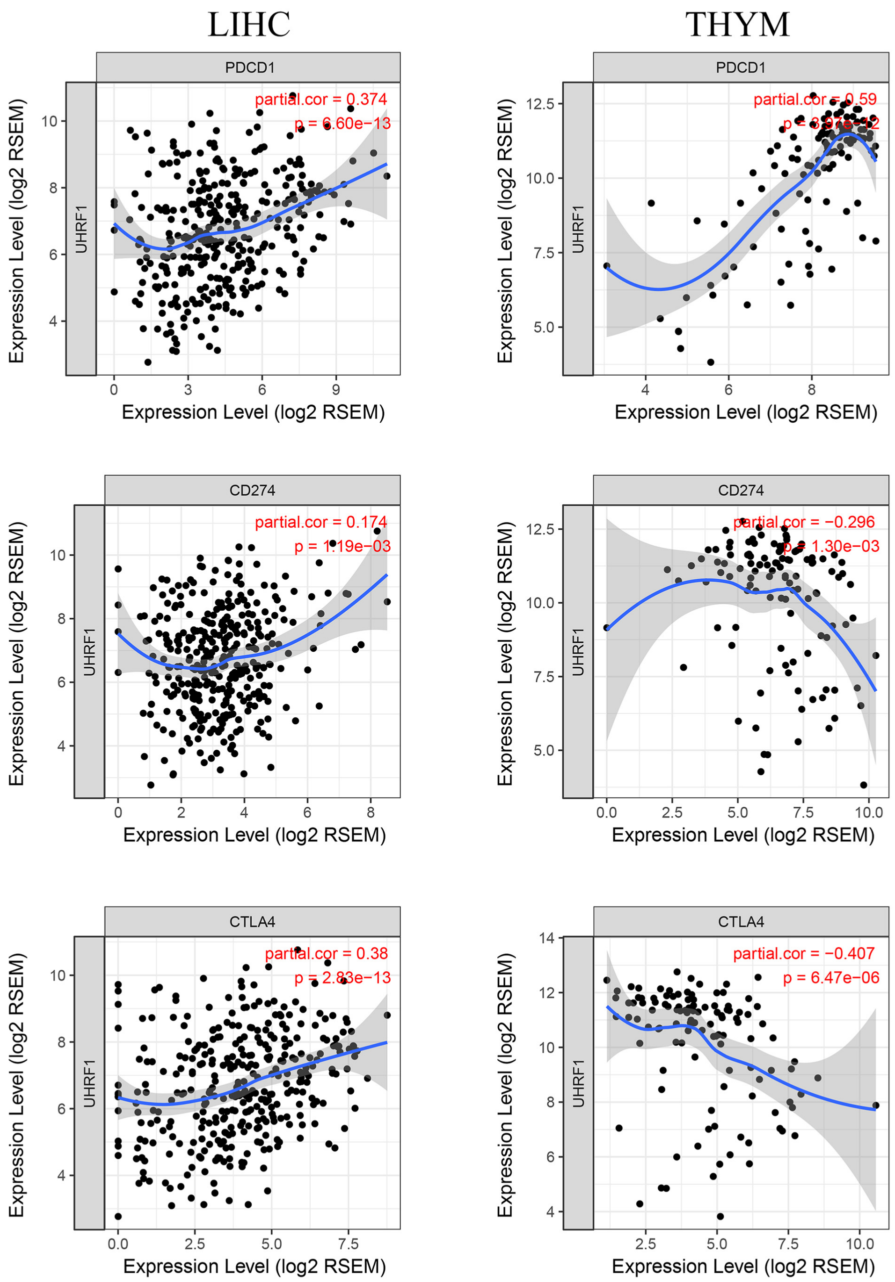

Supplementary Figure 3. Correlation analysis between UHRF1 expression and PD-1, PD-L1 and CTLA-4.
