# Supplementary material for: A disparate role of RP11-424C20.2/UHRF1 axis through control of tumor immune escape in liver hepatocellular carcinoma and thymoma
Source: Aging (Albany NY). 2019 Aug 23;11(16):6422–39. doi: 10.18632/aging.102197 (PMC6738438; doi:10.18632/aging.102197)
Supplement: Supplementary Tables [file aging-11-102197-s001.pdf]

SUPPLEMENTARY TABLES

Supplementary Table 1. Correlation analysis between UHRF1 and biomarkers of monocyte, dendritic cell, Th1 and T cell exhaustion in LIHC and THYM in GEPIA.

Description	Gene markers	LIHC		THYM	
		cor	P	cor	P
Monocyte	CD86	0.4	8.9e-16	−0.36	5.2e-05
	CD115 (CSF1R)	0.32	3.3e-10	−0.3	0.001
Dendritic cell	HLA-DPB1	0.32	5.5e-10	−0.32	0.00051
	HLA-DQB1	0.18	0.00056	−0.16	0.092
	HLA-DRA	0.33	6.9e-11	−0.3	0.0011
	HLA-DPA1	0.25	1.1e-06	−0.27	0.0028
	BDCA-1(CD1C)	0.23	1.2e-05	0.73	0
	BDCA-4(NRP1)	0.3	7.5e-09	−0.32	0.00051
	CD11c (ITGAX)	0.22	1.3e-05	−0.29	0.0014
Th1	T-bet (TBX21)	0.13	0.013	−0.22	0.019
	STAT4	0.16	0.0021	−0.22	0.018
	STAT1	0.22	2.2e-05	−0.3	8e-04
	IFN-γ (IFNG)	0.22	1.4e-05	−0.32	0.00034
	TNF-α (TNF)	0.17	0.0011	−0.28	0.0021
T cell exhaustion	PD-1 (PDCD1)	0.28	4.7e-08	0.65	2.2e-15
	CTLA4	0.21	5.4e-05	−0.26	0.0046
	LAG3	0.29	1.4e-08	−0.32	0.00047
	TIM-3 (HAVCR2)	0.18	0.00069	−0.26	0.0038
	GZMB	0.18	0.00073	−0.26	0.0038

Supplementary Table 2. Correlation analysis between STAT1 and TNF-α, IFN-γ, PD-L1 and CTLA-4 in LIHC and THYM in GEPIA.

Gene	LIHC		THYM	
	cor	P	cor	P
IFN-γ	0.47	0	0.39	1.3e-05
PD-L1 (CD274)	0.24	2.5e-06	0.41	3.5e-06
CTLA-4	0.44	0	0.32	0.00039
